# Supplementary material for: DArTSeq SNP-based markers revealed high genetic diversity and structured population in Ethiopian cowpea [Vigna unguiculata (L.) Walp] germplasms
Source: PLoS One. 2020 Oct 8;15(10):e0239122. doi: 10.1371/journal.pone.0239122 (PMC7544073; doi:10.1371/journal.pone.0239122)
Supplement: S1 Table — (DOCX) [file pone.0239122.s001.docx]

S1. Table. Collection region, number of accession from each region and altitudinal ranges of the test cowpea genetic resources

| **Geographic region** | **Zone of**  **collection** | **Districts**  **covered** | **No. of collections** | **Names of genotypes** | **Altitudinal range (masl)** |
| --- | --- | --- | --- | --- | --- |
| Amhara | North shewa, East Gojjam, Wag Hemra, South Wollo, North Wollo, Oromo-Liyu zone | Ankober, Hulet Eju Enese, Ataye, Shewarobit, Enebsie Sar midir, Abergele, Dese Robit, Sekota, Haike, Lasta, Bati, Kalu, Raya Kobo | 70 | CP7, CP9, CP13, CP14, CP19, CP22, CP23, CP25, CP26, CP27, CP30, CP69, CP70, CP71, CP76, CP85, CP86, CP137, CP138, CP139, CP140, CP165, CP166, CP167, CP168, CP169, CP170, CP171, CP172, CP173, CP174, CP175, CP176, CP177, CP178, CP179, CP180, CP181, CP190, CP191, CP192, CP193, CP194, CP231, CP232, CP233, CP234, CP235, CP236, CP237, CP240, CP241, CP242, CP244, CP245, CP246, CP247, CP248, CP249, CP250, CP253, CP254, CP255, CP256, CP257, CP258, CP259, CP262, CP263, CP264 | 1104-2609 |
| Gambella | Anuak, Nuer | Itang special woreda, Abobo, Gog, Gambella zuria | 59 | CP2, CP3, CP4, CP8, CP10, CP11, CP18, CP24, CP35, CP43, CP47, CP48, CP52, CP77, CP90, CP91, CP92, CP93, CP94, CP95, CP121, CP122, CP123, CP124, CP125, CP200, CP201, CP202, CP206, CP207, CP208, CP209, CP210, CP230, CP265, CP266, CP267, CP268, CP269, CP270, CP271, CP272, CP273, CP274, CP275, CP276, CP279, CP280, CP355, CP356, CP357, CP358, CP359, CP360, CP363, CP364, CP365, CP366, CP367, | 303- 570 |
| Oromia | West Welega, East Hararge, East Shewa, West Harerge, | Dangur, Biya Aaale, Sayo, Guba koricha, Babilea, Gursum, Boset, Adea, odabultune, Habra, Chero zuria, Girera, Fedis | 49 | CP1, CP12, CP33, CP34, CP36, CP40, CP41, CP46, CP49, CP61, CP62, CP64, CP65, CP66, CP67, CP68, CP82, CP83, CP84, CP89, CP98, CP99, CP100, CP126, CP127, CP128, CP129, CP130, CP182, CP183, CP188, CP189, CP195, CP196, CP197, CP198, CP204, CP205, CP220, CP221, CP222, CP223, CP224, CP225, CP226, CP260, CP261, CP277, CP278 | 1280-2439 |
| SNNPRS | Gamo gofa, Bench Maji, Alaba, sheka, [Kembata Tembaro](https://en.wikipedia.org/wiki/Kembata_Tembaro_Zone), Dirashe, south omo, Wolayta, Konso, | Arbaminch zuria, Konso special woreda, Dirashe special woreda, Bena Tsemay, South Ari, Humbo, Sodo zuria, Abela Faracho, Yeki, Alaba | 92 | CP5, CP6, CP17, CP37, CP42, CP44, CP50, CP51, CP53, CP54, CP55, CP58, CP59, CP60, CP63, CP87, CP88, CP96, CP97, CP101, CP102, CP103, CP104, CP105, CP106, CP107, CP108, CP109, CP110, CP111, CP112, CP113, CP114, CP115, CP116, CP117, CP118, CP119, CP120, CP131, CP132, CP134, CP135, CP136, CP155, CP156, CP157, CP158, CP184, CP185, CP186, CP187, CP199, CP211, CP212, CP213, CP214, CP215, CP216, CP217, CP219, CP227, CP228, CP229, CP281, CP282, CP283, CP284, CP285, CP331,CP332, CP333, CP334, CP335, CP336, CP337, CP338, CP339, CP340, CP341, CP342, CP343, CP344, CP345, CP346, CP347, CP348, CP349, CP350, CP352, CP353, CP354 | 1200-1773 |
| Tigray | [Central Tigray](https://en.wikipedia.org/wiki/Mehakelegnaw_Zone), [East Tigray](https://en.wikipedia.org/wiki/Misraqawi_Zone), [North West Tigray](https://en.wikipedia.org/wiki/Semien_Mi%27irabawi_Zone), [South Tigray](https://en.wikipedia.org/wiki/Debubawi_Zone), [South East Tigray](https://en.wikipedia.org/wiki/Debub_Misraqawi_Zone), [West Tigray](https://en.wikipedia.org/wiki/Mi%27irabawi_Zone), [Mekele](https://en.wikipedia.org/wiki/Mek%27ele) |  | 40 | CP15, CP16, CP20, CP21, CP28, CP31, CP32, CP38, CP39, CP56, CP57, CP72, CP73, CP74, CP75, CP141, CP142, CP143, CP144, CP145, CP146, CP147, CP148, CP149, CP150, CP151, CP152, CP153, CP154, CP159, CP160, CP161, CP162, CP163, CP238, CP239, CP243, CP251, CP252, CP286 | 1490-2175 |
| Breeding line | Introduced from International Institute for Tropical Agriculture (IIAT) | | 40 | TVU – 7144, TVU – 7146, TVU – 7148, TVU – 7149, TVU – 14568, TVU – 15548, IT 11D-24-40, IT 10K-817-1, IT 10K-827-11, IT 97K-556-4, IT 07K-243-1-2, IT 89KD-288, IT - 00K - 901-5, IT - 0T - 03L-2046-2, IT - 86D – 378, IT - 87D – 1137, IT - 87D – 721, IT - 89KD, IT - 93K - 556-4, IT - 93K - 452-1, IT - 93K - 619-1, IT - 93K - 2046-1, IT - 93K - 293-2-2, IT - 95K - 1095-4A, IT - 95K - 268-1-4, IT - 96 D – 604, IT - 96 D – 610, IT - 96 D – 719, IT - 960 – 604, IT - 97K - 449-38, IT - 97K - 499-38, IT - 97K - 356-1, IT - 97K - 568-18, IT - 97K - 569-9, IT - 98K - 1111-1, IT - 98K - 506-1, IT - 99K – 1060, IT - 99K - 316-2, MEL-NURL-96-3, IT 09K-456 |  |
| Released varieties |  |  | 7 | Bole, TVU, Black Eye Bean, Bekur, Kenketi, Asebot, Asrat |  |
