# Supplementary material for: DArTSeq SNP-based markers revealed high genetic diversity and structured population in Ethiopian cowpea [Vigna unguiculata (L.) Walp] germplasms
Source: PLoS One. 2020 Oct 8;15(10):e0239122. doi: 10.1371/journal.pone.0239122 (PMC7544073; doi:10.1371/journal.pone.0239122)
Supplement: S2 Table — (DOCX) [file pone.0239122.s002.docx]

S2 Table. Distribution and genetic diversity parameters of 6498 SNPs measured in a set of 357 cowpea genotypes

| **Chr. No** | **No of SNPs** | **MAF** | **AN** | **He** | **Ho** | **PIC** | **F_IS_** |
| --- | --- | --- | --- | --- | --- | --- | --- |
| 1 | 552 | 0.79 | 2.74 | 0.31 | 0.12 | 0.26 | 0.60 |
| 2 | 463 | 0.76 | 2.78 | 0.34 | 0.12 | 0.29 | 0.63 |
| 3 | 849 | 0.77 | 2.77 | 0.33 | 0.12 | 0.28 | 0.63 |
| 4 | 564 | 0.77 | 2.83 | 0.34 | 0.13 | 0.29 | 0.63 |
| 5 | 606 | 0.79 | 2.81 | 0.31 | 0.12 | 0.26 | 0.61 |
| 6 | 557 | 0.74 | 2.78 | 0.36 | 0.13 | 0.30 | 0.63 |
| 7 | 740 | 0.77 | 2.76 | 0.33 | 0.12 | 0.28 | 0.63 |
| 8 | 531 | 0.78 | 2.80 | 0.32 | 0.12 | 0.27 | 0.63 |
| 9 | 538 | 0.80 | 2.77 | 0.30 | 0.11 | 0.25 | 0.63 |
| 10 | 510 | 0.77 | 2.80 | 0.34 | 0.12 | 0.28 | 0.65 |
| 11 | 588 | 0.77 | 2.79 | 0.34 | 0.13 | 0.28 | 0.62 |
| **Mean** | 591 | 0.77 | 2.78 | 0.33 | 0.12 | 0.28 | 0.63 |
| **SE** | 33.27 | 0.005 | 0.008 | 0.005 | 0.002 | 0.005 | 0.004 |

MAF, major allele frequency; AN, number of allele; Ho, observed heterozygosity; He, gene diversity; PIC, Polymorphic Information Content; FIS, inbreeding coefficient.
