# Supplementary material for: DArTSeq SNP-based markers revealed high genetic diversity and structured population in Ethiopian cowpea [Vigna unguiculata (L.) Walp] germplasms
Source: PLoS One. 2020 Oct 8;15(10):e0239122. doi: 10.1371/journal.pone.0239122 (PMC7544073; doi:10.1371/journal.pone.0239122)
Supplement: Attachment — Submitted filename: Declaration_ SK.pdf [file pone.0239122.s005.pdf]

M.B. BRADLEY
P.O. Box 37326
Faerie Glen
Pretoria 0043

072 369 5149

DECLARATION ON EDITING

**Customer**: Selamawit Ketema	**Date**
**Student no:**	2020/02/10

Document submitted for editing: Article

DArTSeq SNP-based markers revealed high genetic diversity and structured population in Ethiopian cowpea [Vigna unguiculata (L.) Walp] germplasms

The above article was submitted to me for language editing, which was completed on 10 February 2020.

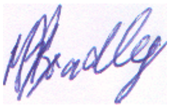

M.B. BRADLEY (MA) - Language editor - Cell no 072 3695 149
