# Supplementary material for: DArTSeq SNP-based markers revealed high genetic diversity and structured population in Ethiopian cowpea [Vigna unguiculata (L.) Walp] germplasms
Source: PLoS One. 2020 Oct 8;15(10):e0239122. doi: 10.1371/journal.pone.0239122 (PMC7544073; doi:10.1371/journal.pone.0239122)
Supplement: Attachment — Submitted filename: Combined response to Reviewers_August 13.docx [file pone.0239122.s006.docx]

Rebuttal letter in response to the comments forwarded by the reviewers on manuscript number, PONE-D-19-32934 [DArTSeq SNP-based markers revealed high genetic diversity and structured population in Ethiopian cowpea [Vigna unguiculata (L.) Walp] germplasms].

Dear Editor and Reviewers

We are grateful for the reviewers who have been reviewing our manuscript during this COVID -19 pandemic time. We are also thankful for the valuable comments given to improve our manuscript. We would also like to seek apology for the delayed response for this paper, this was due to communication gap among the authors worsened by the pandemic situation and the internet restriction by the Ethiopian gouvernement. The reviewer’s comments have helped us to improve the manuscript and we have tried our best to address all the comments given by the reviewers. The changes made in the manuscript are shown in track in the manuscript. The response for each comment, along with the query of the reviewer is summarize as below.

Sincerely,

The Authors

**Revision notes**

**Reviewer 1 Comments:**

**Comment 1.** This manuscript is to describe the diversity of cowpea in Ethiopian, why the author added breeding line and varieties which is not collected from Ethiopian? I agree and recommend adding a certain amount of breeding lines for diversity analysis, however, the source of the materials does not associate with the main idea of the article, it will cause confusion in the analysis. If the author implied or thought those breeding lines were all from Ethiopia, why compare breeding lines with all Ethiopian landraces as two independent groups in Table 1 and 2?

**Response 1.**). One of our objectives is to see if the landraces collected from different regions of Ethiopia maintained and used by the local farmers are different from the cowpea genotypes collected from IITA or developed by breeding program in Ethiopia. The inclusion of the breeding lines is driven by the fact that the cowpea-breeding program, being dependent on the introduced cultivars imported and/or from the International Institute of Tropical Agriculture (IITA), would represent exotic/external germplasm outside Ethiopia. Our goal in comparing breeding lines/improved variety from IITA with Ethiopian landraces is to see the allelic richness of these two groups and hence develop a better germplasm management strategy and for use for breeding in Ethiopia and worldwide. In addition, to mitigate the bias that can be introduced by the difference in number of samples, we have compared the improved varieties with each of the subpopulation that constitute the whole population for landraces.

**Comment 2.** How did the authors pick up the 314 landraces from whole germplasms? Are these materials representative of the entire germplasm pool? Theoretically, diversity analysis must collect all local landraces, but considering the research cost. The researchers will select some highly diverse materials from all the collections to represent the whole germplasm pool. Please clarify whether these materials have such qualifications. My suggestion is that if the 314 landraces are part of germplasm, the author should provide the phenotype of all germplasm resources and prove the representativeness of the selected material; if this material is the entire germplasm of Ethiopia, it is also desirable to conduct analysis and statistics on the phenotype of this material.

**Response 2.** The 314 landraces represent the entire collection of cowpea germplasm in Ethiopia. From this study we planned to only phenotype the core collection extensively for several key and economically important traits to cut on cost usually associated with efficient broad genetic stock/germplasm characterization.

**Comment 3.** How did the authors screen the 6498 markers from the 20276 SNPs? The main problem of GBS is missing and false data, please clarify the quality control of genotype. In my suggestion, it is also better to prove the distribution of the 6498 SNPs in each chromosome, because of the character of GBS sequencing, the average distance of markers is too much different from the real situation.

**Response 3.** The comment is accepted, and one paragraph was inserted in material and methods under the section “SNPs calling and data filtering” regarding data filtering to respond to the request query. Data filtering conditions were included as follow: SNP markers with high proportion of missing data (>10%), individuals who have high rates of genotype missingness (>10%) and rare SNPs with <5% minor allele frequency (MAF) were discarded from further analysis using R software (version 2.8.3). The most informative SNPs were selected based on a threshold PIC value equal or higher than 0.2. Finally 357 cowpea genotypes and 6498 (32%) of SNP markers were maintained for further analysis.

In addition, the distribution of SNPs per chromosomes was provided in S2 Table. The number of SNPs ranges from 463 on chromosome 2 to 849 on chromosome 3 with a mean of 591 SNPs per chromosome

**Comment 4. Please re-make Fig 2, 5 and 6 to improve the resolution, if the authors could**

**Response 4**. The comment is accepted, and new captures were done to improve the figure quality subsequently. The figures were also validated through the PLOS one PACE requirements.

**Comment 5.** Except for the flaw of the scientific part, my main concerns are:
The germplasm in this manuscript was only from one country, which makes it of limited reference value to researchers worldwide. Please elaborate on the implications of this study for researchers worldwide. Overall, although the diversity analysis of this study was relatively integrated, I did not see any innovation, depth understanding, or thinking about diversity research from the conclusions and perspectives.

To sum up, I am conservative and cautious about the publication of this manuscript immediately, unless the authors can find new research ideas or impressive conclusion with a more thorough discussion and understanding from current results. My suggestion is that, since the author has mentioned the establishment of a core collection, it is better to build the “core germplasm” for Ethiopia based on the existing data combining with the phenotype of whole the germplasm. This project is more important than an only diversity study, and its results are more valuable for reference over the world.

Response 5. The queries and suggestion of comment 5 has been addressed as follows and included in the conclusion to show the importance of the current study and its potential use worldwide

- Although different reports indicated that East Africa including Ethiopia is one of the centers of origin, center of diversity or secondary center of diversity there was no cowpea collection neither any characterization to such extent in Ethiopia cowpea landraces. Though this collection was only done in Ethiopia, the result of this study has shown the existence of genetic diversity in the landraces and it expected that these germplasms might have unharnessed potential for future breeding owing different trait for cowpea improvement. Thus, these germplasms can be used globally for cowpea future breeding.

Innovation, depth understanding, or thinking about diversity research from the conclusions and perspectives.

- Worldwide, germplasm collection has been done for most crops for a long period, and it is assumed as we already had enough collection. However, this study could show, as we have not still collected the germplasm resource of the world. Thus, this study sheds light on how we could still have several germplasm in most crops that needs collection, preservation, and use for future breeding. In this study in the future, the collected germplasm will be thoroughly phenotype, and used for cowpea improvement for the target traits. A core set of diagnostic makers can be developed to serve worldwide purpose

My suggestion is that, since the author has mentioned the establishment of a core collection, it is better to build the “core germplasm” for Ethiopia based on the existing data combining with the phenotype of whole the germplasm,

- The suggestion for establishing core collection is well taken and included in the manuscript as shown in the introduction, material method, result, discussion and conclusion. However, we wouldn’t be able to phenotype the whole collection due to budget limitation, hence we will be focusing on phenotyping extensively the core collection for several traits

**Reviewer 2 Comments:**

**Comment 1.** The authors forgot to include line numbers in the manuscript, which make it difficult to mention typographical errors. A correction of the text is recommended.

**Response 1** Comment accepted and line numbers included

**Comment 2.** In the abstract replace 357 cowpea “germplasms” by 357 cowpea accessions

**Response 2**. Comment accepted and correction made according to the suggestion

**Comment 3.** In the introduction, the first sentence needs correction; also, there is a lack of consensus on where in Africa cowpea was domesticated. Authors should take that in account.

**Response 3**. Comment accepted and the sentence was rephrased as “Cowpea *[Vigna unguiculata (L.) Walp*., 2n = 2x = 22*]* originated and was domesticated in Africa though the exact location of origin of domestication is still a matter of speculation and different authors suggest different areas in Africa, Northeastern Africa including Ethiopia (1–4), Central Africa (5), South Africa (6), and West Africa (5,7,8). (Line 45 to line 48)

**Comment 4**. Methods section: a brief description of the sequencing method is recommended.

**Response 4.** A brief description of the GBS protocol as developed and implemented by DArT was added to the Material and Method under the section “DNA extraction and sequencing”

**Comment 5**. Authors should provide detailed information on the SNP calling approach, SNP filtering thresholds as well as the filtering of the accessions.

**Response 5.** One paragraph was inserted in Material and Methods section with respect to data filtering under a new title “SNP calling and data filtering”

**Comment 6.** Population structure analysis: I would recommend a membership coefficient of ≥ 0.70.

Response 6. The comment accepted and membership coefficient of ≥ 0.70 were used and the results were corrected accordingly.

**Comment 7**. Authors should also replace the figures with high resolution figures.

**Response 7**. The comment is accepted, and the figures have been replaced with figures with higher resolution quality.

**Response to reviewer comments, August 05**

The reviewer comments are shown below in black and the response by the authors is provided as response to reviewers in italics

Dear Dr Yao,

Thank you for submitting your manuscript entitled "DArTSeq SNP-based markers revealed high genetic diversity and structured population in Ethiopian cowpea [Vigna unguiculata (L.) Walp] germplasms" to PLOS ONE. Your manuscript files have been checked in-house but before we can proceed we need you to address the following issues:

1) Thank you for updating your data availability statement. You note that your data are available within the Supporting Information files, but no such files have been included with your submission. At this time we ask that you please upload your minimal data set as a Supporting Information file, or to a public repository such as Figshare or Dryad.

 Please also ensure that when you upload your file you include separate captions for your supplementary files at the end of your manuscript.

As soon as you confirm the location of the data underlying your findings, we will be able to proceed with the review of your submission.

***Response 1 to reviewers****: The genotyping data set was included as supporting information and a caption was added at the end of the manuscript under the title: S3: Genotyping supporting data obtained after filtering used for the study*

2) Please amend your list of authors on the manuscript to ensure that each author is linked to an affiliation.
We note that you have included affiliation numbers 1-6 and *, ¶ however only affiliations 1-6 and * have authors linked to them. Please amend affiliation ¶ to link an author to it or remove if added in error.

***Response 2 to reviewers****: The affiliation “¶” was added by mistake and was removed from the author list*

3) Please include a copy of Table 4 which you refer to in your text on page 10.

***Response 3 to reviewers****: Table 4 and Table 6 should read the same. The numbering as Table 6 came from the fact that S1 and S2 tables were previously included in the manuscript body as Table 4 and Table 5 before been moved as supplementary files. Then the actual Table 6 was amended as Table 4 in the table caption and in the text body.*

**Response to reviewer comments, August 13**

The reviewer comments are shown below in black and the response by the authors is provided as response to reviewers in italics and blue

Dear Dr Yao

Thank you for submitting your manuscript entitled "DArTSeq SNP-based markers revealed high genetic diversity and structured population in Ethiopian cowpea [Vigna unguiculata (L.) Walp] germplasms" to PLOS ONE.  Your manuscript files have been checked in-house but before we can proceed we need you to address the following issues:

1. We note that Figure 1 in your submission contain [map/satellite] images which may be copyrighted. All PLOS content is published under the Creative Commons Attribution License (CC BY 4.0), which means that the manuscript, images, and Supporting Information files will be freely available online, and any third party is permitted to access, download, copy, distribute, and use these materials in any way, even commercially, with proper attribution. For these reasons, we cannot publish previously copyrighted maps or satellite images created using proprietary data, such as Google software (Google Maps, Street View, and Earth). For more information, see our copyright guidelines: <http://journals.plos.org/plosone/s/licenses-and-copyright>.

***Response to reviewer****: The map shown in figure 1 was constructed by the authors using the GPS coordinates taken during collection. The construction was done using the free software DIVA-GIS as described by Hijmans et al., (2012). The material and method section was slightly modified to include how the map of Figure 1 was generated. The data set used to generate the map of the collection sites was added to the manuscript as supplementary files (S1 file). The former S3 table was re-numbered S2 file and changed within the text accordingly*
